# Estrogen Contributions to Microvascular Dysfunction Evolving to Heart Failure With Preserved Ejection Fraction

**DOI:** 10.3389/fendo.2019.00442

**Published:** 2019-07-03

**Authors:** Ariane A. Sickinghe, Suzanne J. A. Korporaal, Hester M. den Ruijter, Elise L. Kessler

**Affiliations:** Laboratory of Experimental Cardiology, University Medical Center Utrecht, Utrecht, Netherlands

**Keywords:** heart failure with preserved ejection fraction, microvascular dysfunction, sex differences, estrogens, endothelial dysfunction, impaired angiogenesis, (perivascular) fibrosis, capillary rarefaction

## Abstract

Heart failure with preserved ejection fraction (HFpEF) is a syndrome involving microvascular dysfunction. No treatment is available yet and as the HFpEF patient group is expanding due to the aging population, more knowledge on dysfunction of the cardiac microvasculature is required. Endothelial dysfunction, impaired angiogenesis, (perivascular) fibrosis and the pruning of capillaries (rarefaction) may all contribute to microvascular dysfunction in the heart and other organs, e.g., the kidneys. The HFpEF patient group consists mainly of post-menopausal women and female sex itself is a risk factor for this syndrome. This may point toward a role of estrogen depletion after menopause in the development of HFpEF. Estrogens favor the ratio of vasodilating over vasoconstricting factors, which results in an overall lower blood pressure in women than in men. Furthermore, estrogens improve angiogenic capacity and attenuate (perivascular) fibrosis formation. Therefore, we hypothesize that the drop of estrogen levels after menopause contributes to myocardial microvascular dysfunction and renders post-menopausal women more vulnerable for heart diseases that involve the microvasculature. This review provides a detailed summary of molecular targets of estrogen, which might guide future research and treatment options.

## Introduction

Cardiovascular disease (CVD) is the most common cause of death worldwide with 17.9 million deaths annually^1^. As the world adopts more westernized lifestyles, the incidence of CVD risk factors, like obesity and diabetes, increases. There are known sex differences in prevalence, incidence, risk factors, and prognosis of different CVDs (1). When corrected for age, in Europe, CVD is responsible for 49% of the deaths among women and 41% among men, causing death in over 4 million people every year (2). The incidence of CVDs is higher in men before the age of 50 than in pre-menopausal women. However, after menopause, CVD incidence in women rises and eventually exceeds CVD incidence in men (3). Furthermore, men more often die of ischemic heart disease, while women more often die from stroke and heart failure (HF) (3).

### Epidemiology and Comorbidities for HFpEF

Although HF was originally associated with a reduced left ventricular (LV) ejection fraction (HFrEF), it now seems that nearly half of the HF patients (45–50%) have a preserved ejection fraction (HFpEF) (4). In both sexes, the incidence of HFpEF, and HFrEF increases with age. However, HFpEF incidence is higher in women at any given age, especially in post-menopausal women. In contrast, HFrEF incidence is higher in men at any given age (4–6).

HFrEF develops following an ischemic event involving the bigger vessels of the heart, e.g., a myocardial infarction (MI), that leads to the reduced contraction of the ventricles (7). Comorbidities, like diabetes, obesity, and smoking are seen in both HFrEF, and HFpEF, but there are evident sex differences in the degree of association of some comorbidities for HF and underlying CVDs; hypertension, atrial fibrillation, female sex, and age have a stronger correlation with HFpEF^1^. Comorbidities and diseases present in HFpEF in combination with higher age (after menopause) and estrogen levels affect several pathophysiologic pathways, which will be illustrated in this review.

HFpEF is characterized by left ventricular diastolic dysfunction (LVDD) (8), and develops gradually suggested to involve the microvasculature of the heart (6, 9). In contrast to post-menopausal women, pre-menopausal women seem to be protected from HFpEF and other CVDs, which points toward the influence of estrogens in the development of microvascular dysfunction. Insights into the molecular basis of diseases affecting the microvasculature of the heart are lacking, but given that the prevalence of HFpEF is increasing, this information is crucial (4). Several pathological processes like endothelial dysfunction, impaired angiogenesis, (perivascular) fibrosis, and blood vessel rarefaction are estrogen-mediated and contribute to microvascular dysfunction. Expanding our knowledge on the role of estrogens on these pathophysiological processes may guide new treatment options for cardiac syndromes like HFpEF.

This review will provide an in-depth summary of the impact of estrogens on pathophysiological processes in which the cardiac microvasculature is involved.

### The Vascular Tone

The most important component of vascular function is the regulation of the vascular tone, which is coordinated by the ability of vascular smooth muscle (VSM) cells to react to changes in blood flow. The vascular tone is strictly regulated by vasoconstricting and vasodilating factors that can be systemically and locally derived and is coordinated by Ca^2+^ influx into the cells. If the vascular tone is chronically increased, hypertension develops, and as a consequence this contributes to the development of CVDs and cardiac remodeling (10, 11). Vascular tone can be regulated by the nervous system, which stimulates vasoconstriction through α-adrenergic receptors and vasodilatation through β-adrenergic receptors.

In the heart, the vascular tone is mostly controlled by energy demands of the myocardium, which are sensed in the microvasculature. Hypoxia activates the systemic renin-angiotensin (Ang II)-aldosterone-system (RAAS), in which Ang II induces VSM constriction. Sustained RAAS activation can lead to extensive collagen deposition (fibrosis) and stiffening of blood vessels, which culminates into an increased vascular tone (12). The vascular endothelium appears to be a key player in the regulation of the vascular tone: (sex) hormones and shear force trigger the endothelium to secrete factors that influence contractility of VSM cells (13).

### Endothelial Dysfunction and Dysregulation of the Vascular Tone

HFpEF patients often present with dysregulation of the vascular tone involving a disturbed balance of secretion of vasoconstricting and -dilating factors by the endothelium of the microvasculature. Vasodilation almost always involves NO produced from L-arginine by three NO synthases of which endothelial NO synthase (eNOS) is the most important for vascular tone regulation (11). NO elevates cyclic GMP (cGMP) concentrations in VSM cells by stimulation of soluble guanylyl cyclase (sGC) activity. Elevated cGMP levels activate protein kinase G (PKG), which diminishes Ca^2+^ levels in the cell through multiple mechanisms, and consequently vasorelaxation is induced (14). Reduced availability and/or responsiveness to NO hinders the vasculature to accustom to constriction/dilatation in response to blood flow changes (15).

**Figure 1:**
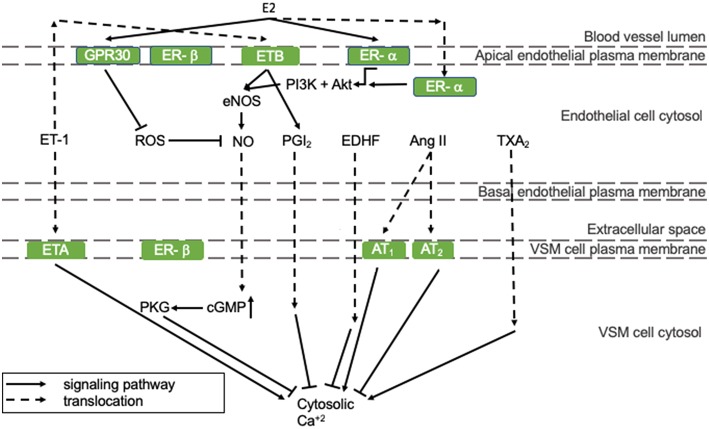
The regulation of endothelial function. All presented pathways affect the cytosolic Ca^2+^ concentration in VSM cells by regulating endothelial function, which determines constriction/relaxation. Solid arrows indicate stimulation and lines with a flattened end indicate inhibition. Dashed lines indicate translocation of the molecule. Green squares are receptors. Ang II, angiotensin-II; AT, angiotensin-II receptor; cGMP, cyclic guanosine monophosphate; GPR30, G-protein coupled estrogen receptor 30; E2, 17β-estradiol; EDHF, endothelial-derived hyperpolarizing factor; eNOS, endothelial nitric oxide synthase; ER, estrogen receptor; ET, endothelin-1 receptor; ET-1, endothelin-1; MAPK/ERK, mitogen-associated protein kinase/extracellular signal-regulated kinases; NO, nitric oxide; PGI_2_, prostacyclin; PI3K, phosphoinositide 3-kinase; PKG, protein kinase G; ROS, reactive oxygen species; TXA_2_, thromboxane A_2_.

Vasodilating pathways can be modulated by estrogens, as depicted in Figure 1. In detail, 17β-estradiol (E2) can positively regulate eNOS activity and thereby NO production by binding to the estrogen receptors (ER)-α and -β that are both present in endothelial cells (represented in the mid-section of Figure 1) (16). Especially endothelial ER-α has cardioprotective properties. Although estrogen receptors are mostly present in the nucleus, where they act as transcription factors, a small pool of ERs has been reported to lay in the vicinity of the plasma membrane, where they stimulate eNOS activity involving protein kinases PI3K and Akt (right section of Figure 1) (17). ER-α activation prevents fatty streak formation by decreasing lipoprotein deposition acting on local inflammation and immune regulation of the early atherosclerotic plaque. This effect is achieved independently of eNOS (18). As the HFpEF phenotype is associated with a proinflammatory state (19), this supports our hypothesis that E2 prevents HFpEF in pre-menopausal women. Alongside endothelial expression, ER-α and -β are also present in VSM cells, where they inhibit VSM cell proliferation in female, but not in male rats, through p38/MAPK signaling (20). In VSM cells, the expression of ER-α, and -β does not differ significantly between male and female rats (21). However, ER-β is up-regulated more in women following pressure overload, and this has been correlated with an inhibition of Ang II-induced hypertrophy in female mice (22, 23). In addition, it protects against reperfusion-induced arrhythmias and inflammation following ischemia in rodents (24).

Other vasodilating factors are prostacyclin (PGI_2_), endothelial-derived hyperpolarizing factor (EDHF), and adenosine. E2 stimulates PGI2 production through ER-α and cyclooxygenase (COX)-1 signaling (25). EDHF can compensate for NO loss under pathological conditions and some studies indicate that EDHF-mediated vasodilatation is more prevalent in pre-menopausal women than in post-menopausal women and men, which is reviewed by Villar et al. (26). This might protect pre-menopausal women from impaired vasodilatation and the development of HFpEF. Adenosine receptor expression has been shown to be altered upon E2 administration in an *in vitro* model. However, the implication of this observation in vascular function has not been investigated yet (27).

Vasoconstricting factors are endothelin-1 (ET-1), Ang II, thromboxane A_2_ (TXA_2_), and reactive oxygen species (ROS) (11). The influence of E2 on Ang II generation and has not been reported. However, in a I/R mouse model, E2 influences the ratio of angiotensin II receptor (ANGTR)1/ANGTR2 in favor of the first, which was associated with protection against I/R injury (28). E2 does not influence TXA_2_ production in an *in vitro* model (25). Although the effects of E2 on ET-1 expression and activity have not been reported, the expression pattern of ET-1 receptors in men renders them more vulnerable for ET-1 induced vasoconstriction compared to women (29).

ROS, such as superoxide, hydroxyl radical, lipid peroxyl radical, and alkoxyl radicals increase the influence of inflammation, diabetes, obesity, and age on endothelial function and thereby NO bioavailability (11). ROS levels are lower in women than men due to differences in phosphorylation patterns of mitochondrial proteins, e.g., aldehyde dehydrogenase (ALDH)2. In an ischemia/reperfusion (I/R) rat model, it was found that ALDH2, an enzyme that detoxifies ROS-generated aldehyde products, had an increased phosphorylation and activity in females compared to males. This was associated with less I/R injury in female mice (30).

E2 can effect ROS levels: E2 decreases oxidative stress by upregulating mitochondrial enzyme, e.g., manganese superoxide dismutase, levels and activity (31). Moreover, ROS formation can be inhibited by E2-G protein-coupled estrogen receptor 30 (GPR30) signaling, which is illustrated by the observation that GRP30 deficiency results in oxidative stress in ovariectomized rats (32). Furthermore, GPR30 signaling seems to be more prevalent in females than males, protecting females from oxidative stress (represented in the left section of Figure 1) (33). In a HFpEF mouse model, the GPR30 agonist G1 was able to abolish abnormal cardiac structure, fibrosis formation, and LVDD (34). This highlights the importance of GPR30 signaling in the pathophysiology of HFpEF. Furthermore, G1 might provide an interesting therapy option for HFpEF patients.

## Impaired Angiogenesis

**Figure 2:**
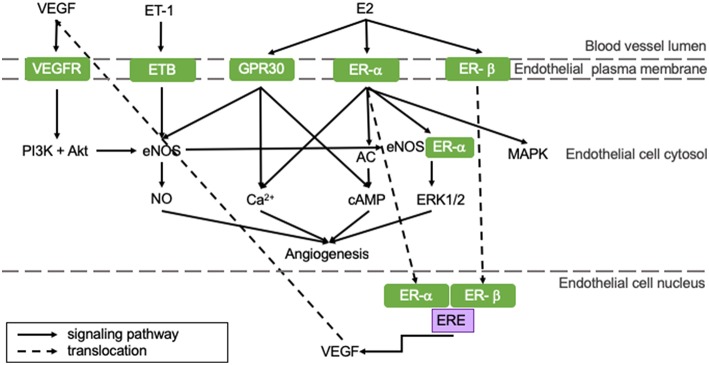
E2 positively influences angiogenic pathways resulting in an increased angiogenic capacity of pre-menopausal women potentially protecting them from HFpEF. All represented pathways positively affect angiogenesis in the heart. Estrogens influence pro-angiogenetic processes, rendering women less susceptible for impaired angiogenesis. This potentially protects pre-menopausal women from HFpEF. AC, adenylate cyclase; cAMP, cyclic adenosine monophosphate; E2, 17β-estradiol; eNOS, endothelial nitric oxide synthase; ER, estrogen receptor; ERE, estrogen responsive element; ET, endothelin-1 receptor; ET-1, endothelin-1; GPR30, G protein-coupled estrogen receptor 30; MAPK, mitogen-activated protein kinase; NO, nitric oxide; PI3K, phosphoinositide 3-kinase; VEGF, vascular endothelial growth factor; VEGFR, vascular endothelial growth factor receptor.

Angiogenesis is the process in which blood vessels sprout from pre-existing capillaries to meet the increased oxygen demands of developing or damaged tissue. It is essential for physiological processes, like growth, responses to sustained exercise, the estrous cycle, wound healing and aging, as well as for recovery from pathological processes like hypertrophy and ischemia (35). Angiogenesis is induced by a number of myocardium-derived factors, of which vascular endothelial growth factor (VEGF) is the most prominent. Other regulators include angiopoietin-1 and−2 (ANGPT-1 and−2), fibroblast growth factor (FGF), transforming growth factor (TGF), and platelet-derived growth factor (PDGF). These factors regulate angiogenesis through various signaling pathways on genomic and non-genomic levels and their role in myocardial angiogenesis has been extensively reviewed in the literature (36–39). The most important pathways that are influenced by E2 are shown in Figure 2.

E2 can stimulate angiogenesis: upon E2 binding, ER-α and -β translocate to the nucleus and bind the estrogen response element (ERE) of the *VEGF* gene, thereby upregulating transcription of VEGF and stimulation of angiogenesis (represented in the right section of Figure 2) (40). VEGF stimulates eNOS, which in turn activates tyrosine and PI3 kinases (left section of Figure 2). This regulates NO production and release by endothelial cells (41). Especially ER-α activation stimulates angiogenesis through both genomic and non-genomic processes (42). Non-genomic processes involve rapid changes in activities of adenylate cyclase (AC), mitogen-activated protein kinase (MAPK), PI3K and eNOS or in concentrations of cytoplasmic Ca^2+^. GPR30-activation can induce angiogenesis via non-genomic processes, like Ca^2+^ influx, cyclic adenosine monophosphate (cAMP) synthesis or PI3K activation, which is presented in the right section of Figure 2 (43). Furthermore, ER-α and eNOS assembly initiates ERK1/2 signaling, thereby promoting reendothelialization (44).

Pre-menopausal women have higher baseline levels of E2 compared to men and post-menopausal women. Therefore, they may have a better angiogenic capacity following MI (42). Furthermore, hypoxia can induce angiogenesis involving sirtuins (SIRT), proteins responsible for maintaining mitochondrial function and cellular metabolism. They have been shown to be key regulators in the coupling of hypoxia-induced angiogenesis and its levels decrease with age (45). SIRT3 transcription can be increased by E2-ER-β signaling in an *in vitro* model, thereby promoting hypoxia-induced angiogenesis (46). The importance of SIRT was indicated in a sirtuin-deleted mouse model, in which angiogenesis was impaired. This led to the development of LVDD (47). Taken all this together, we postulate that the reduced estrogen levels in post-menopausal women contribute to an impaired hypoxia-angiogenesis coupling evolving to LVDD and the development of HFpEF.

## (Perivascular) Fibrosis Formation

Perivascular fibrosis is the formation of fibrosis around blood vessels. While replacement fibrosis is more prevalent in HFrEF, HFpEF is associated with perivascular fibrosis formation in the microvasculature independent of epicardial stiffening (48–50). Estrogens can influence fibrosis formation through several signaling pathways. A recent study shows that rat cardiac fibroblast ER-α activation by E2 leads to inhibition of collagen I and III production in females, while E2 binding to ER-β promotes collagen production in males (51). However, another study shows that increased levels of ER-β after MI protect from inflammation and fibrosis formation in female mice (52). These studies do not have to be conflictive seeing that ER-α and -β dimerization inhibits collagen deposition, and an increased ER-β concentration could amplify this (51). Interestingly, androgens can influence cardiac fibrosis formation by upregulation of TGF-β, which is known to induce extracellular matrix (ECM) deposition predisposing men to cardiac fibrosis (53). Sex differences in cardiac structural remodeling and fibrosis have been extensively reviewed and are beyond the scope of this review.

**Figure 3:**
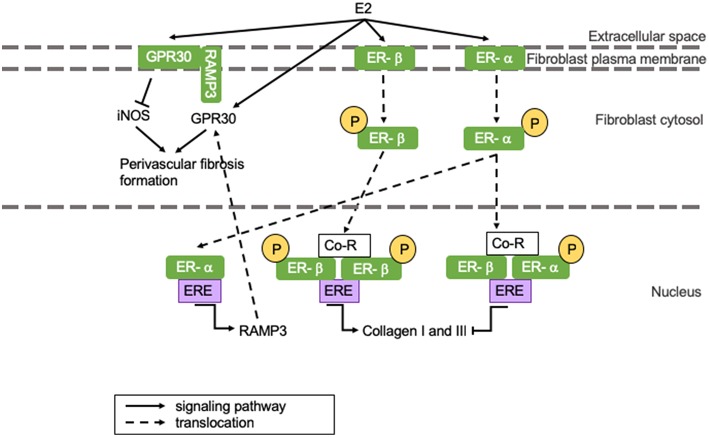
The influence of E2 on (perivascular) fibrosis formation. (Perivascular) fibrosis formation is attenuated in women through E2-induced inhibition of collagen production. However, E2 receptor dimerization promotes fibrosis formation in men. GPR30 localization in men results in perivascular fibrosis formation, while GPR30 localization at the plasma membrane inhibits fibrosis formation in women. Yellow circles indicated with a “P” represent phosphorylated serine residues. ER-α is phosphorylated at Ser-118, while ER-β is phosphorylated at Ser108. Ang II, angiotensin II; AR, androgen receptor; ARE, androgen responsive element; ATR, angiotensin II receptor; Co-R, co-receptor; E2, 17β-estradiol; ER, estrogen receptor; ERE, ααestrogen responsive element; GPR30, G protein coupled receptor 30; iNOS, inducible nitric oxide synthase; RAMP3, receptor activity modifying protein 3; TGF-β, transforming growth factor-β.

Perivascular fibrosis and cardiac hypertrophy can be reduced by E2-induced GPR30 activation, which results in the suppression of inducible NOS (iNOS) activity (represented in the left section of Figure 3) (34). iNOS activity is minimal under physiological conditions, but it is activated during infections and chronic inflammation, where it continuously produces NO (11). iNOS impairs vasoconstriction by activating sGC, but simultaneously reduces vasodilatation by limiting tetrahydrobiopterin (BH_4_) availability for eNOS, thus inducing vascular dysfunction (54). When GPR30 is located at the plasma membrane, it appears to have cardioprotective effects, whilst GPR30 located at the cytosol is associated with perivascular fibrosis formation (55). GPR30 is held at the plasma membrane by receptor activity modifying protein 3 (RAMP3) (55). RAMP3 expression is regulated by E2, and as a consequence, women present with more GPR30 located at the plasma membrane than men (represented in the left section of Figure 3) (56). This is represented in Figure 3. We postulate that the drop of estrogen levels after menopause induces perivascular fibrosis formation involving above-mentioned mechanisms leading to HFpEF.

## Capillary Rarefaction

Rarefaction is the decrease of capillary density, causing hypoxia in mice and rats (57). It is associated with hypertension (58, 59), hypertrophy (60), diabetes (61), and aging in multiple tissues in both men and women (62). Coronary microvascular rarefaction was shown to be a prominent cause of HFpEF in male and female patients (63, 64). However, mechanisms underlying coronary rarefaction are not fully understood, but are likely comparable to those in other circulatory organs, such as the kidneys. Inflammation, dysregulation of angiogenic molecules, and pericyte loss are phenomena underlying kidney rarefaction. E2 influences some of these pathologic processes and we postulate that these mechanisms could also play a role in coronary rarefaction contributing hypoxia in HFpEF, as will be described in the following section. However, capillary rarefaction may also be the result of cardiomyocyte death leading to a decreased oxygen need.

### Lessons From Renal Rarefaction

Upon renal transplantation, capillary dilatation and rarefaction are strongly correlated with intracapillary inflammation (65). It is known that TNF-α mediated inflammation affects vascular endothelial cells, which can result in apoptosis *in vitro* (66). Endothelial apoptosis leads to pruning of blood vessels in humans (67). TNF-α is a cytokine mediating the inflammatory response and promoting apoptosis by inhibition of Akt-mediated cell survival (68). E2 can inhibit TNF-α induced apoptosis by binding to its ER-β-receptor, which induces Akt phosphorylation and Notch1 expression, thereby promoting cell survival (66). Furthermore, women show lower LV expression of pro-inflammatory genes during pressure-overload than men (69). Consequently, pre-menopausal women might be protected from inflammation-induced apoptosis of vascular endothelial cells and resulting rarefaction.

Shear force is required for endothelial cell survival, through Akt phosphorylation (70). Furthermore, shear force stimulates the production of pro-angiogenic factors, such as NO and VEGF. When shear force is diminished, cell survival and pro-angiogenic signals are reduced and this results in vessel pruning (71). The role of VEGF dysregulation in kidney rarefaction is extensively reviewed (57). Whether this plays a role in coronary vascular endothelial cell apoptosis in a reaction to changes in shear force still has to be determined.

### Role of Pericytes

Pericytes are perivascular cells that stabilize the vascular wall and maintain vascular quiescence and integrity. During kidney and lung fibrosis, TGF-β stimulates pericytes to detach from the vascular wall and differentiate into myofibroblasts, contributing to myocardial fibrosis (72, 73). The loss of pericytes in the perivascular region leads to destabilization of capillaries, capillary dysfunction and, ultimately, rarefaction. Although the role of pericytes in lung and kidney fibrosis has been established, the importance of pericytes in ventricular remodeling during HFpEF has not been fully established. There are implications that pericytes could play a role in coronary capillary rarefaction. Pericyte recruitment is regulated by the ratio of ANGPT-1 vs.-2, molecules that are secreted by mesenchymal and endothelial cells (74). Both molecules can bind the endothelial Tie-2 receptor. Binding of ANGPT-1 attracts pericytes and stimulates vessel assembly and maturation, whereas ANGPT-2 impairs pericyte recruitment. The latter was shown to induce vessel rarefaction in a tumor mouse model (74). Thrombospondin-1 (TSP-1) can disturb the ANGPT-1/-2 ratio in favor of ANGPT-2, thereby inducing capillary rarefaction in a diabetic mouse model (61). The influence of E2 on TSP-1 levels and responsiveness or ANGPT-1 and-2 stimulation have not been reported so far.

## Discussion

As established in this review, sex differences in the molecular mechanisms of endothelial dysfunction, impairment of angiogenesis, (perivascular) fibrosis formation, and capillary rarefaction are abundant and render men more vulnerable for CVDs at younger age than pre-menopausal women. However, after menopause, E2 levels drop and testosterone levels increase in women, which is associated with an elevated risk for CVDs, coronary heart disease (CHD) and HF (3, 75, 76). High E2 levels in post-menopausal women are associated with a lower risk of CHD (77). Post-menopausal women outnumber men in a ratio of 2:1 in CVDs, in which the microvasculature is affected, such as in HFpEF (78). Besides the effects of E2 on pathophysiologic processes described in this review, E2 affects other systemic and cellular processes. First of all, it is postulated that, in reaction to low E2 levels after menopause, RAAS is activated, which increases ROS and decreases NO. This results in an increase of collagen synthesis and LVDD, the main characteristic of HFpEF (6). Moreover, the effects of E2 on mitochondrial metabolism in LVDD have been described in the literature. In short, E2 maintains mitochondrial biogenesis and function through several signaling pathways, involving e.g., peroxisome proliferator-activated receptor (PPAR)α, nuclear respiratory factor (NRF)-1 and AMP-activated protein kinase (AMPK). Loss of E2 is associated with abnormal mitochondrial function, oxidative stress, and LVDD (79). Therefore, we hypothesize that due to the drop of E2 levels after menopause, the protective effects of E2 on the microvasculature involving the above-mentioned mechanisms are strongly diminished, which results in the incidence of HFpEF in the two decades following menopause. Also, non-hormone related causes can be appointed: in an acute I/R four core genotype mouse model, where you can investigate the sex chromosome effect independently of the sex hormone effect, the presence of a second X chromosome made mice more vulnerable for I/R damage. It was suggested that incomplete inactivation of the second X chromosome may result in escaping genes that are constitutively higher expressed in mice with two X chromosomes. This suggests that a second X chromosome by itself has a detrimental effect on the vasculature and the heart (80). If and how sex chromosomes interplay with sex hormones and affect HF subtypes is yet to be determined. Together, these studies suggest that the development of HFpEF is an interplay between several sex-related processes.

### Future Perspectives and Treatment Options

Interestingly, E2 level-restoring therapies, like hormone therapy (HT), show conflicting effects. The Women's Health Initiative (WHI) hormone therapy trial was a large trial designed to investigate the effects of HT in women between 50 and 79 years of age. Although HT was beneficial for the management of menopausal symptoms in healthy women, the use of HT for the prevention of chronic disease was not supported. The risks of HT outweighed the benefits in all age groups (81). In this trial only one dose was used, therefore the smaller Kronos Early Estrogen Prevention Study (KEEPS) and the Early vs. Late Interventional Trial with Estradiol (ELITE) study sought to investigate the effects of different doses and forms of estradiol at several time points around menopause in women without CVD at entry. Although the ELITE study showed that estradiol could slow atherosclerosis progression in post-menopausal women within 6 years after menopause, the KEEPS study did not show beneficial effects on the vasculature. HT, however, did not seem to be harmful for cardiovascular health either (81, 82). Up to now, there is no data on the effects of HT on the development or progression of LVDD or HFpEF and it remains to be investigated if long-term menopausal HT could reduce the development of HFpEF.

Therapies that improve the vascular function of the heart might be promising. Angiogenic therapies have been under investigation during the last 20 years. Although preclinical results are promising, clinical trial results are unambiguous (83). Angiogenic gene therapy seems to be more effective in post-menopausal women than in men and younger women. But overall, angiogenic therapy alone does not improve vascularity significantly in a clinical setting (84). Better definition of patient sub-groups, improvement of targeted delivery and combination with other therapies might improve angiogenic therapy response. The information on sex differences in angiogenesis provided in this review might assist the development of such regenerative therapies, e.g., involving pericytes.

Revascularization therapy using pericytes has shown to be effective in the attenuation of cardiac remodeling. Transplantation of pericytes into the ischemic myocardium improves the capillary density in the heart with 45% compared to non-pericyte injected hearts in mice (85). Pericytes accomplish this effect by upregulating VEGF, PDGF-β, and TGF-β. The beneficial effects of TGF-β are questionable since other studies report that TGF-β induces pericyte differentiation and fibrosis (72, 73). Pericyte transplantation might be effective in the attenuation of cardiac remodeling evolving to HFpEF, but before clinical translation to HFpEF patients can be made more research should be conducted into molecular effects of pericyte transplantation in a HFpEF setting. Furthermore, the long-term efficiency of pericyte transplantation might be limited due to cell longevity and repeated injections could hinder the clinical application. The use of pericytes in regenerative medicine is relatively new and more research should be conducted into phenotyping and function should be conducted.

Inhibition of vascular decay by chemically promoting pericyte localization at the vascular wall could provide an efficient therapy against capillary rarefaction. This could also avoid trans-differentiation of pericytes into myofibroblasts decreasing (peri) vascular fibrosis. Especially in diseases like HFpEF, in which the smallest vessels in the heart are susceptible for decay, vascular stabilization poses an interesting research option. Vascular decay might also be interesting in the search for biomarkers for microvascular dysfunction, which could help detect vascular remodeling preceding CVDs in an early stage with low patient burden. Post-menopausal women suffering from HFpEF are underdiagnosed as efficient microvascular imaging techniques are expensive and time-consuming. The use of a microvascular decay biomarker might help the early detection of HFpEF patients.

## Conclusion

Microvascular dysfunction is a prominent contributor to HFpEF in men and post-menopausal women. Treatment options for these diseases have not been successful so far. There are differences between the sexes and between pre- and post-menopausal women that render pre-menopausal women less vulnerable for microvascular dysfunction and subsequent CVDs. Considering differences in prevalence of the processes mentioned in this review, we hypothesize that capillary rarefaction might be more prevalent in post-menopausal women than in pre-menopausal women and men, thereby rendering them more vulnerable for CVDs associated with microvascular decay, e.g., HFpEF. On a molecular level, suggested therapeutic targets, including GPR30 and ETA. GPR30 agonists and ETA antagonists are promising for improving microvascular function and preventing decay, which might attenuate HFpEF (34, 86).

## Author Contributions

AS and EK conceived and designed the manuscript. AS drafted the manuscript with support of EK, who supervised the work. SK and HdR were involved in revising the manuscript critically for important intellectual content. All authors gave final approval of the manuscript to be published and agreed to be accountable for all aspects of the work.

### Conflict of Interest Statement

The authors declare that the research was conducted in the absence of any commercial or financial relationships that could be construed as a potential conflict of interest.

## Footnotes

^1^http://www.who.int/en/news-room/fact-sheets/detail/cardiovascular-diseases-(cvds).

## Abbreviations

AC: adenylate cyclase
ACS: acute coronary syndromes
AMPK: AMP activated protein kinase
Ang II: angiotensin-II
ANGPT: angiopoietin
AR: androgen receptor
ARE: androgen responsive element
AS: aortic stenosis
ATR: angiotensin-II receptor
BH_4_: tetrahydrobioptin
CAD: coronary artery disease
cAMP: cyclic adenosine monophosphate
cGMP: cyclic guanosine monophosphate
CHD: coronary heart disease
Co-R: co-receptor
COX: cyclooxogenase
CVD: cardiovascular disease
E2: 17β-estradiol
ECM: extracellular matrix
EDHF: endothelial-derived hyperpolarizing factor
EDV: end diastolic volume
eNOS: endothelial nitric oxide synthase
ER: estrogen receptor
ERE: estrogen response element
ET: endothelin-1 receptor
ET-1: endothelin-1
FGF: fibroblast growth factor
GPR30: G protein-coupled estrogen receptor 30
HF: heart failure
HFpEF: heart failure with preserved ejection fraction
HFrEF: heart failure with reduced ejection fraction
HT: hormone replacement therapy
I/R: ischemia/reperfusion
LVDD: left ventricular diastolic dysfunction
MAPK: mitogen-activated protein kinase
MI: myocardial infarction
NO: nitric oxide
NRF: nuclear respiratory factor
PDGF: platelet-derived growth factor
PGI_2_: protacyclin
PI3K: phosphoinositide 3-kinase
PKG: protein kinase G
PPAR: peroxisome proliferator-activated receptor
RAAS: renin-angiotensin-aldosterone-system
RAMP3: receptor activity modifying protein 3
ROS: reactive oxygen species
SCAD: spontaneous coronary artery dissection
sGC: soluble guanylyl cyclase
SIRT: Sirtuin
TGF: transforming growth factor
TNF-α: tumor necrosis factor-α
TNFR: tumor necrosis factor receptor
TSP-1: thrombospondin-1
TXA_2_: thromboxane A_2_
VEGF: vascular endothelial growth factor
VEGFR: vascular endothelial growth factor receptor
VSM: vascular smooth muscle.

## References

[1] Regitz-Zagrosek V Kararigas G. Mechanistic pathways of sex differences in cardiovascular disease. Physiol Rev. (2017) 97:1–37. 10.1152/physrev.00021.2015 27807199

[2] Townsend N Nichols M Scarborough P Rayner M Cardiovascular disease in Europe — epidemiological update 2015. Eur Heart J. (2015) 36:2696–705. 10.1093/eurheartj/ehv428 26306399

[3] Mozaffarian D Benjamin EJ Go AS Arnett DK Blaha MJ Cushman M. Heart disease and stroke statistics-−2016 update. Circulation. (2016) 133:e38–360. 10.1161/CIR.0000000000000350 26673558

[4] Dunlay SM Roger VL Redfield MM. Epidemiology of heart failure with preserved ejection fraction. Nat Rev Cardiol. (2017) 14:591–602. 10.1038/nrcardio.2017.65 28492288

[5] Lam CSP Donal E Kraigher-Krainer E Vasan RS. Epidemiology and clinical course of heart failure with preserved ejection fraction. Eur J Heart Fail. (2011) 13:18–28. 10.1093/eurjhf/hfq121 20685685 PMC3003453

[6] Beale AL Meyer P Marwick TH Lam CSP Kaye DM. Sex differences in cardiovascular pathophysiology why women are overrepresented in heart failure with preserved ejection fraction. Circulation. (2018) 138:198–205. 10.1161/CIRCULATIONAHA.118.034271 29986961

[7] Bloom MW Greenberg B Jaarsma T Januzzi JL Lam CSP Maggioni AP. Heart failure with reduced ejection fraction. Nat Rev Dis Prim. (2017) 3:17058. 10.1038/nrdp.2017.58 28836616

[8] Borlaug BA. The pathophysiology of heart failure with preserved ejection fraction. Nat Rev Cardiol. (2014) 11:507–15. 10.1038/nrcardio.2014.83 24958077

[9] Lee JF Barrett-O'Keefe Z Garten RS Nelson AD Ryan JJ Nativi JN. Evidence of microvascular dysfunction in heart failure with preserved ejection fraction. Heart. (2016) 102:278–84. 10.1136/heartjnl-2015-308403 26567228 PMC4866903

[10] Dai Z Aoki T Fukumoto Y Shimokawa H. Coronary perivascular fibrosis is associated with impairment of coronary blood flow in patients with non-ischemic heart failure. J Cardiol. (2012) 60:416–21. 10.1016/j.jjcc.2012.06.009 22867802

[11] Konukoglu D Uzun H. Endothelial Dysfunction and Hypertension. Cham: Springer (2016). p. 511–40. 10.1007/5584_2016_90 28035582

[12] Murphy E Amanakis G Fillmore N Parks RJ Sun J Sex differences in metabolic cardiomyopathy. Cardiovasc Res. (2017) 113:370–7. 10.1093/cvr/cvx008 PMC5852638 28158412

[13] Shaw J Anderson T. Coronary endothelial dysfunction in non-obstructive coronary artery disease: risk, pathogenesis, diagnosis and therapy. Vasc Med. (2016) 21:146–55. 10.1177/1358863X15618268 26675331

[14] Patel D Lakhkar A Wolin MS. Redox mechanisms influencing cGMP signaling in pulmonary vascular physiology and pathophysiology. Adv Exp Med Biol. (2017) 967:227–40. 10.1007/978-3-319-63245-2_13 29047089 PMC5766272

[15] Heiss C Rodriguez-Mateos A Kelm M. Central role of eNOS in the maintenance of endothelial homeostasis. Antioxid Redox Signal. (2015) 22:1230–42. 10.1089/ars.2014.6158 25330054 PMC4410282

[16] Nevzati E Shafighi M Bakhtian KD Treiber H Fandino J Fathi AR. Estrogen induces nitric oxide production via nitric oxide synthase activation in endothelial cells. In: Fandino J Marbacher S Fathi A-R Muroi C Keller E, editors. Neurovascular Events After Subarachnoid Hemorrhage. Cham: Springer International Publishing (2015). p. 141–5. 10.1007/978-3-319-04981-6_24 25366614

[17] Gourdy P Guillaume M Fontaine C Adlanmerini M Montagner A Laurell H. Estrogen receptor subcellular localization and cardiometabolism. Mol Metab. (2018) 15:56–69. 10.1016/j.molmet.2018.05.009 29807870 PMC6066739

[18] Elhage R Arnal J-F Pieraggi M-T Duverger N Fiévet C Faye J-C 17β-Estradiol prevents fatty streak formation in apolipoprotein E–deficient mice. Arterioscler Thromb Vasc Biol. (1997) 17:2679–84. 10.1161/01.ATV.17.11.2679 9409242

[19] Paulus WJ Tschöpe C. A novel paradigm for heart failure with preserved ejection fraction: comorbidities drive myocardial dysfunction and remodeling through coronary microvascular endothelial inflammation. J Am Coll Cardiol. (2013) 62:263–71. 10.1016/j.jacc.2013.02.092 23684677

[20] Pellegrini M Bulzomi P Lecis M Leone S Campesi I Franconi F. Endocrine disruptors differently influence estrogen receptor β and androgen receptor in male and female rat VSMC. J Cell Physiol. (2014) 229:1061–8. 10.1002/jcp.24530 24347325

[21] Hogg ME Vavra AK Banerjee MN Martinez J Jiang Q Keefer LK. The role of estrogen receptor α and β in regulating vascular smooth muscle cell proliferation is based on sex. J Surg Res. (2012) 173:e1–10. 10.1016/j.jss.2011.09.021 22099601 PMC3286876

[22] Nordmeyer J Eder S Mahmoodzadeh S Martus P Fielitz J Bass J. Upregulation of myocardial estrogen receptors in human aortic stenosis. Circulation. (2004) 110:3270–5. 10.1161/01.CIR.0000147610.41984.E8 15533858

[23] Pedram A Razandi M Lubahn D Liu J Vannan M Levin ER. Estrogen inhibits cardiac hypertrophy: role of estrogen receptor-β to inhibit calcineurin. Endocrinology. (2008) 149:3361–9. 10.1210/en.2008-0133 18372323 PMC2453079

[24] Wang Y Wang Q Zhao Y Gong D Wang D Li C. Protective effects of estrogen against reperfusion arrhythmias following severe myocardial ischemia in rats. Circ J. (2010) 74:634–43. 10.1253/circj.CJ-09-0223 20173305

[25] Sobrino A Oviedo PJ Novella S Laguna-Fernandez A Bueno C García-Pérez MA. Estradiol selectively stimulates endothelial prostacyclin production through estrogen receptor-α. J Mol Endocrinol. (2010) 44:237–46. 10.1677/JME-09-0112 20110403

[26] Villar IC Hobbs AJ Ahluwalia A. Sex differences in vascular function: implication of endothelium-derived hyperpolarizing factor. J Endocrinol. (2008) 197:447–62. 10.1677/JOE-08-0070 18492811

[27] Mohamadi A Aghaei M Panjehpour M. Estrogen stimulates adenosine receptor expression subtypes in human breast cancer MCF-7 cell line. Res Pharm Sci. (2018) 13:57–64. 10.4103/1735-5362.220968 29387112 PMC5772082

[28] Xue Q Xiao D Zhang L. Estrogen regulates angiotensin ii receptor expression patterns and protects the heart from ischemic injury in female rats. Biol Reprod. (2015) 93:6. 10.1095/biolreprod.115.129619 25972014 PMC4706310

[29] Gohar EY Giachini FR Pollock DM Tostes RC. Role of the endothelin system in sexual dimorphism in cardiovascular and renal diseases. Life Sci. (2016) 159:20–9. 10.1016/j.lfs.2016.02.093 26939577 PMC4992599

[30] Lagranha CJ Deschamps A Aponte A Steenbergen C Murphy E. Sex differences in the phosphorylation of mitochondrial proteins result in reduced production of reactive oxygen species and cardioprotection in females. Circ Res. (2010) 106:1681–91. 10.1161/CIRCRESAHA.109.213645 20413785 PMC3127199

[31] Stirone C Duckles SP Krause DN Procaccio V. Estrogen increases mitochondrial efficiency and reduces oxidative stress in cerebral blood vessels. Mol Pharmacol. (2005) 68:959–65. 10.1124/mol.105.014662 15994367

[32] Wang H Sun X Lin MS Ferrario CM Van Remmen H Groban L. G protein-coupled estrogen receptor (GPER) deficiency induces cardiac remodeling through oxidative stress. Transl Res. (2018) 199:39–51. 10.1016/j.trsl.2018.04.005 29758174 PMC6151279

[33] Debortoli AR Rouver W do N Delgado NTB Mengal V Claudio ERG. GPER modulates tone and coronary vascular reactivity in male and female rats. J Mol Endocrinol. (2017) 59:171–80. 10.1530/JME-16-0117 28733475

[34] Wang X Tan Y Xu B Lu L Zhao M Ma J. GPR30 Attenuates myocardial fibrosis in diabetic ovariectomized female rats: role of iNOS signaling. DNA Cell Biol. (2018) 37:821–30. 10.1089/dna.2018.4208 30227089

[35] Barnabas O Wang H Gao X-M. Role of estrogen in angiogenesis in cardiovascular diseases. J Geriatr Cardiol. (2013) 10:377–82. 10.3969/j.issn.1671-5411.2013.04.008 24454332 PMC3888921

[36] Jeansson M Gawlik A Anderson G Li C Kerjaschki D Henkelman M. Angiopoietin-1 is essential in mouse vasculature during development and in response to injury. J Clin Invest. (2011) 121:2278–89. 10.1172/JCI46322 21606590 PMC3104773

[37] Andrae J Gallini R Betsholtz C. Role of platelet-derived growth factors in physiology and medicine. Genes Dev. (2008) 22:1276–312. 10.1101/gad.1653708 18483217 PMC2732412

[38] Chen J-X Zeng H Reese J Aschner JL Meyrick B. Overexpression of angiopoietin-2 impairs myocardial angiogenesis and exacerbates cardiac fibrosis in the diabetic *db/db* mouse model. Am J Physiol Circ Physiol. (2012) 302:H1003–12. 10.1152/ajpheart.00866.2011 22180648 PMC3322731

[39] Dobaczewski M Chen W Frangogiannis NG. Transforming growth factor (TGF)-β signaling in cardiac remodeling. J Mol Cell Cardiol. (2011) 51:600–6. 10.1016/j.yjmcc.2010.10.033 21059352 PMC3072437

[40] Buteau-Lozano H Ancelin M Lardeux B Milanini J Perrot-Applanat M. Transcriptional regulation of vascular endothelial growth factor by estradiol and tamoxifen in breast cancer cells: a complex interplay between estrogen receptors alpha and beta. Cancer Res. (2002) 62:4977–84. 12208749

[41] Papapetropoulos A García-Cardeña G Madri JA Sessa WC. Nitric oxide production contributes to the angiogenic properties of vascular endothelial growth factor in human endothelial cells. J Clin Invest. (1997) 100:3131–9. 10.1172/JCI119868 9399960 PMC508526

[42] Mahmoodzadeh S Leber J Zhang X Jaisser F Messaoudi S Morano I. Cardiomyocyte-specific estrogen receptor alpha increases angiogenesis, lymphangiogenesis and reduces fibrosis in the female mouse heart post-myocardial infarction. J Cell Sci Ther. (2014) 5:153. 10.4172/2157-7013.1000153 24977106 PMC4070011

[43] Trenti A Tedesco S Boscaro C Trevisi L Bolego C Cignarella A. Estrogen, angiogenesis, immunity and cell metabolism: solving the puzzle. Int J Mol Sci. (2018) 19:859. 10.3390/ijms19030859 29543707 PMC5877720

[44] Billon A Lehoux S Lam Shang Leen L Laurell H Filipe C Benouaich V The estrogen effects on endothelial repair and mitogen-activated protein kinase activation are abolished in endothelial nitric-oxide (NO) synthase knockout mice, but not by no synthase inhibition by N-nitro-l-arginine methyl ester. Am J Pathol. (2008) 172:830–8. 10.1016/j.niox.2008.06.209 18276789 PMC2258270

[45] He X Zeng H Chen J-X. Ablation of SIRT3 causes coronary microvascular dysfunction and impairs cardiac recovery post myocardial ischemia. Int J Cardiol. (2016) 215:349–57. 10.1016/j.ijcard.2016.04.092 27128560 PMC4890543

[46] Panza S Santoro M De Amicis F Morelli C Passarelli V D'Aquila P. Estradiol via estrogen receptor beta influences ROS levels through the transcriptional regulation of SIRT3 in human seminoma TCam-2 cells. Tumor Biol. (2017) 39:101042831770164. 10.1177/1010428317701642 28459202

[47] Maizel J Xavier S Chen J Lin CHS Vasko R Goligorsky MS. Sirtuin 1 ablation in endothelial cells is associated with impaired angiogenesis and diastolic dysfunction. Am J Physiol Circ Physiol. (2014) 307:H1691–704. 10.1152/ajpheart.00281.2014 25239805 PMC4269697

[48] Pandey A Khan H Newman AB Lakatta EG Forman DE Butler J. Arterial stiffness and risk of overall heart failure, heart failure with preserved ejection fraction, and heart failure with reduced ejection fraction. Hypertension. (2017) 69:267–74. 10.1161/HYPERTENSIONAHA.116.08327 27993954 PMC5828168

[49] Alex L Russo I Holoborodko V Frangogiannis NG. Characterization of a mouse model of obesity-related fibrotic cardiomyopathy that recapitulates features of human heart failure with preserved ejection fraction. Am J Physiol Circ Physiol. (2018) 315:H934–49. 10.1152/ajpheart.00238.2018 30004258 PMC6230908

[50] Paulus WJ Dal Canto E Distinct myocardial targets for diabetes therapy in heart failure with preserved or reduced ejection fraction. JACC Hear Fail. (2018) 6:1–7. 10.1016/j.jchf.2017.07.012 29284577

[51] Dworatzek E Mahmoodzadeh S Schriever C Kusumoto K Kramer L Santos G. Sex-specific regulation of collagen I and III expression by 17β-Estradiol in cardiac fibroblasts: role of estrogen receptors. Cardiovasc Res. (2018) 115:315–27. 10.1093/cvr/cvy185 30016401 PMC6933535

[52] Zhang J-B Guo C-L. Protective effect and mechanism of estrogen receptor β on myocardial infarction in mice. Exp Ther Med. (2017) 14:1315–20. 10.3892/etm.2017.4628 28810592 PMC5526156

[53] Kong P Christia P Frangogiannis NG. the pathogenesis of cardiac fibrosis. Cell Mol Life Sci. (2014) 71:549–74. 10.1007/s00018-013-1349-6 23649149 PMC3769482

[54] Gunnett CA Lund DD McDowell AK Faraci FM Heistad DD. Mechanisms of inducible nitric oxide synthase–mediated vascular dysfunction. Arterioscler Thromb Vasc Biol. (2005) 25:1617–22. 10.1161/01.ATV.0000172626.00296.ba 15933248

[55] Lenhart PM Broselid S Barrick CJ Leeb-Lundberg LMF Caron KM. G-protein-coupled receptor 30 interacts with receptor activity-modifying protein 3 and confers sex-dependent cardioprotection. J Mol Endocrinol. (2013) 51:191–202. 10.1530/JME-13-0021 23674134 PMC3724340

[56] Wang H Zhao Z Lin M Groban L. Activation of GPR30 inhibits cardiac fibroblast proliferation. Mol Cell Biochem. (2015) 405:135–48. 10.1007/s11010-015-2405-3 25893735 PMC4449333

[57] Afsar B Afsar RE Dagel T Kaya E Erus S Ortiz A. Capillary rarefaction from the kidney point of view. Clin Kidney J. (2018) 11:295–301. 10.1093/ckj/sfx133 29988260 PMC6007395

[58] Ciuffetti G Schillaci G Innocente S Lombardini R Pasqualini L Notaristefano S. Capillary rarefaction and abnormal cardiovascular reactivity in hypertension. J Hypertens. (2003) 21:2297–303. 10.1097/00004872-200312000-00018 14654750

[59] Triantafyllou A Anyfanti P Triantafyllou G Zabulis X Aslanidis S Douma S. Impaired metabolic profile is a predictor of capillary rarefaction in a population of hypertensive and normotensive individuals. J Am Soc Hypertens. (2016) 10:640–6. 10.1016/j.jash.2016.04.007 27265366

[60] Oka T Akazawa H Naito AT Komuro I. Angiogenesis and cardiac hypertrophy: maintenance of cardiac function and causative roles in heart failure. Circ Res. (2014) 114:565–71. 10.1161/CIRCRESAHA.114.300507 24481846

[61] Gonzalez-Quesada C Cavalera M Biernacka A Kong P Lee D-W Saxena A. Thrombospondin-1 induction in the diabetic myocardium stabilizes the cardiac matrix in addition to promoting vascular rarefaction through angiopoietin-2 upregulation. Circ Res. (2013) 113:1331–44. 10.1161/CIRCRESAHA.113.302593 24081879 PMC4408537

[62] Faber JE Zhang H Lassance-Soares RM Prabhakar P Najafi AH Burnett MS. Aging causes collateral rarefaction and increased severity of ischemic injury in multiple tissues. Arterioscler Thromb Vasc Biol. (2011) 31:1748–56. 10.1161/ATVBAHA.111.227314 21617137 PMC3141082

[63] Mohammed SF Hussain S Mirzoyev SA Edwards WD Maleszewski JJ Redfield MM. Coronary microvascular rarefaction and myocardial fibrosis in heart failure with preserved ejection fraction. Circulation. (2015) 131:550–9. 10.1161/CIRCULATIONAHA.114.009625 25552356 PMC4324362

[64] Zeng H Chen J-X. Microvascular rarefaction and heart failure with preserved ejection fraction. Front Cardiovasc Med. (2019) 6:15. 10.3389/fcvm.2019.00015 30873415 PMC6403466

[65] Li X Sun Q Zhang M Xie K Chen J Liu Z. Capillary dilation and rarefaction are correlated with intracapillary inflammation in antibody-mediated rejection. J Immunol Res. (2014) 2014:1–10. 10.1155/2014/582902 24741607 PMC3987932

[66] Fortini F Vieceli Dalla Sega F Caliceti C Aquila G Pannella M Pannuti A. Estrogen receptor β-dependent Notch1 activation protects vascular endothelium against tumor necrosis factor α (TNFα)-induced apoptosis. J Biol Chem. (2017) 292:18178–91. 10.1074/jbc.M117.790121 28893903 PMC5672041

[67] Gobé G Browning J Howard T Hogg N Winterford C Cross R. Apoptosis occurs in endothelial cells during hypertension-induced microvascular rarefaction. J Struct Biol. (1997) 118:63–72. 10.1006/jsbi.1996.3835 9087915

[68] Ohkawara H Ishibashi T Sugimoto K Ikeda K Ogawa K Takeishi Y. Membrane type 1–matrix metalloproteinase/akt signaling axis modulates TNF-α-induced procoagulant activity and apoptosis in endothelial cells. Ushio-Fukai M, editor. PLoS ONE. (2014) 9:e105697. 10.1371/journal.pone.0105697 25162582 PMC4146507

[69] Kararigas G Dworatzek E Petrov G Summer H Schulze TM Baczko I. Sex-dependent regulation of fibrosis and inflammation in human left ventricular remodelling under pressure overload. Eur J Heart Fail. (2014) 16:1160–7. 10.1002/ejhf.171 25287281

[70] Dimmeler S Assmus B Hermann C Haendeler J Zeiher AM. Fluid shear stress stimulates phosphorylation of Akt in human endothelial cells: involvement in suppression of apoptosis. Circ Res. (1998) 83:334–41. 10.1161/01.RES.83.3.334 9710127

[71] Ballermann BJ Obeidat M. Tipping the balance from angiogenesis to fibrosis in CKD. Kidney Int Suppl. (2014) 4:45–52. 10.1038/kisup.2014.9 26312149 PMC4536966

[72] Humphreys BD Lin S-L Kobayashi A Hudson TE Nowlin BT Bonventre JV Fate tracing reveals the pericyte and not epithelial origin of myofibroblasts in kidney fibrosis. Am J Pathol. (2010) 176:85–97. 10.2353/ajpath.2010.090517 20008127 PMC2797872

[73] Wang N Deng Y Liu A Shen N Wang W Du X. Novel mechanism of the pericyte-myofibroblast transition in renal interstitial fibrosis: core fucosylation regulation. Sci Rep. (2017) 7:16914. 10.1038/s41598-017-17193-5 29209018 PMC5717002

[74] Fagiani E Lorentz P Kopfstein L Christofori G. Angiopoietin-1 and−2 exert antagonistic functions in tumor angiogenesis, yet both induce lymphangiogenesis. Cancer Res. (2011) 71:5717–27. 10.1158/0008-5472.CAN-10-4635 21778249

[75] Bui AL Horwich TB Fonarow GC. Epidemiology and risk profile of heart failure. Nat Rev Cardiol. (2011) 8:30–41. 10.1038/nrcardio.2010.165 21060326 PMC3033496

[76] Bhatnagar P Wickramasinghe K Wilkins E Townsend N. Trends in the epidemiology of cardiovascular disease in the UK. Heart. (2016) 102:1945–52. 10.1136/heartjnl-2016-309573 27550425 PMC5256396

[77] Zhao D Guallar E Ouyang P Subramanya V Vaidya D Ndumele CE. Endogenous sex hormones and incident cardiovascular disease in post-menopausal women. J Am Coll Cardiol. (2018) 71:2555–66. 10.1016/j.jacc.2018.01.083 29852978 PMC5986086

[78] Florijn BW Bijkerk R van der Veer EP van Zonneveld AJ. Gender and cardiovascular disease: are sex-biased microRNA networks a driving force behind heart failure with preserved ejection fraction in women? Cardiovasc Res. (2018) 114:210–25. 10.1093/cvr/cvx223 29186452

[79] Li S Gupte AA. The role of estrogen in cardiac metabolism and diastolic function. Methodist Debakey Cardiovasc J. (2017) 13:4–8. 10.14797/mdcj-13-1-4 28413575 PMC5385797

[80] Li J Chen X McClusky R Ruiz-Sundstrom M Itoh Y Umar S. The number of X chromosomes influences protection from cardiac ischaemia/reperfusion injury in mice: one X is better than two. Cardiovasc Res. (2014) 102:375–84. 10.1093/cvr/cvu064 24654234 PMC4030514

[81] Hodis HN Mack WJ Henderson VW Shoupe D Budoff MJ Hwang-Levine J. Vascular effects of early versus late postmenopausal treatment with estradiol. N Engl J Med. (2016) 374:1221–31. 10.1056/NEJMoa1505241 27028912 PMC4921205

[82] Miller VM Naftolin F Asthana S Black DM Brinton EA Budoff MJ. The kronos early estrogen prevention study (KEEPS): what have we learned? Menopause. (2019). 10.1097/GME.0000000000001326. [Epub ahead of print]. 31453973 PMC6738629

[83] Kaski JC Consuegra-Sanchez L. Evaluation of ASPIRE trial: a phase III pivotal registration trial, using intracoronary administration of Generx (Ad5FGF4) to treat patients with recurrent angina pectoris. Expert Opin Biol Ther. (2013) 13:1749–53. 10.1517/14712598.2013.827656 23957658

[84] Ylä-Herttuala S Bridges C Katz MG Korpisalo P Angiogenic gene therapy in cardiovascular diseases: dream or vision? Eur Heart J. (2017) 38:ehw547 10.1093/eurheartj/ehw547 PMC5837788 28073865

[85] Chen C-W Okada M Proto JD Gao X Sekiya N Beckman SA. Human pericytes for ischemic heart repair. Stem Cells. (2013) 31:305–16. 10.1002/stem.1285 23165704 PMC3572307

[86] Samad MA Kim UK Kang JJ Ke Q Kang PM. Endothelin A receptor antagonist, atrasentan, attenuates renal and cardiac dysfunction in Dahl salt-hypertensive rats in a blood pressure independent manner. PLoS ONE. (2015) 10:e0121664. 10.1371/journal.pone.0121664 25775254 PMC4361570

